# Targeted Delivery of Amoxicillin to C. trachomatis by the Transferrin Iron Acquisition Pathway

**DOI:** 10.1371/journal.pone.0150031

**Published:** 2016-02-26

**Authors:** Jun Hai, Nawal Serradji, Ludovic Mouton, Virginie Redeker, David Cornu, Jean-Michel El Hage Chahine, Philippe Verbeke, Miryana Hémadi

**Affiliations:** 1 ITODYS, Interfaces, Traitements, Organisation et Dynamique des Systèmes, Université Paris Diderot, Sorbonne Paris Cité, CNRS-UMR 7086, 15 rue Jean-Antoine de Baïf, 75205 Paris Cedex 13, France; 2 Paris-Saclay Institute of Neuroscience, CNRS-UMR 9197, 1 avenue de la Terrasse, 91190 Gif-sur-Yvette, France; 3 Service d’Identification et de Caractérisation des Protéines, CNRS-UMR 9198, 1 avenue de la Terrasse, 91190 Gif-sur-Yvette, France; 4 UMR 1149 Inserm, Université Paris Diderot, Sorbonne Paris Cité, ERL-CNRS 8252, Faculté de Médecine, site Bichat, 16 rue Henri Huchard, 75018 Paris, France; State University of Maringá/Universidade Estadual de Maringá, BRAZIL

## Abstract

Weak intracellular penetration of antibiotics makes some infections difficult to treat. The Trojan horse strategy for targeted drug delivery is among the interesting routes being explored to overcome this therapeutic difficulty. *Chlamydia trachomatis*, as an obligate intracellular human pathogen, is responsible for both trachoma and sexually transmitted diseases. *Chlamydia* develops in a vacuole and is therefore protected by four membranes (plasma membrane, bacterial inclusion membrane, and bacterial membranes). In this work, the iron-transport protein, human serum-transferrin, was used as a Trojan horse for antibiotic delivery into the bacterial vacuole. Amoxicillin was grafted onto transferrin. The transferrin-amoxicillin construct was characterized by mass spectrometry and absorption spectroscopy. Its affinity for transferrin receptor 1, determined by fluorescence emission titration [K_affTf-amox_ = (1.3 ± 1.0) x 10^8^], is very close to that of transferrin [4.3 x 10^8^]. Transmission electron and confocal microscopies showed a co-localization of transferrin with the bacteria in the vacuole and were also used to evaluate the antibiotic capability of the construct. It is significantly more effective than amoxicillin alone. These promising results demonstrate targeted delivery of amoxicillin to suppress *Chlamydia* and are of interest for *Chlamydiaceae* and maybe other intracellular bacteria therapies.

## Introduction

*Chlamydia trachomatis* (C. trachomatis), as an obligate intracellular human pathogen of the *Chlamydiaceae* family, is responsible for the most common sexually transmitted bacterial infection and is the leading cause of preventable blindness [1]. *Chlamydia* genital infection is very common in sexually active young people. When not treated, it can lead to severe complications including endometriosis, salpingitis, pelvic inflammatory syndromes, pelvic pain and chronic ectopic pregnancies [2–4]. *Chlamydiaceae* are Gram-negative intracellular bacteria that grow and multiply in a parasitophorous vacuole during a two-phase developmental cycle [5]. Infection is initiated by the binding of elementary bodies (EBs) to eukaryotic host cells. A few hours after their internalization, a parasitophorous vacuole forms near the nucleus of the host cell. Metabolically inactive EBs differentiate into reticulate bodies (RBs), non-infectious but metabolically active forms of *Chlamydiaceae*. RBs replicate before then re-differentiating to EBs. The inclusion expands and bursts, releasing the EBs that go on to infect other surrounding cells [5,6]. The infectious EBs are metabolically inert, unable to replicate DNA, to transcribe RNA or to translate proteins. Thus, unlike RB, EB is not affected by antibiotics targeting either bacterial DNA synthesis (fluoroquinolone) or protein translation (macrolides, tetracycline) processes. Therefore, only antibiotics that penetrate cells will be effective against C. trachomatis [7]. However, RB is protected within the host cell by four lipid bilayers (the plasma membrane, the inclusion membrane and the double membrane of the bacterium). This low accessibility to RB and the low hydrophobicity of certain antibiotics may explain the partial ineffectiveness of antibiotic therapy [8,9,10] and, unfortunately, no anti-*Chlamydia trachomatis* vaccine is yet available. To prevent infection relapses, new anti-chlamydial drugs and/or efficient drug carriers are needed.

Iron is essential for the growth and development of both animal cells and prokaryotes. Apotransferrin (ApoTf) is a bilobal (N- and C-lobes) single chain of about 700 amino acids that binds two molecules of Fe^3+^ with high affinity (K_aff_ = 10^23^) [11]. In the bloodstream of mammals, the iron-loaded form of transferrin (holotransferrin, Tf) binds to transferrin receptor 1 (R1). R1 is a 190 kDa homodimeric protein arranged in two subunits linked by two disulfide bridges. It has two domains: an ectodomain directed toward the biological fluid and an endodomain anchored in the plasma membrane [12,13]. Tf interacts with the ectodomain of R1 to form an adduct, Tf-R1, which is internalized in the cytoplasm through clathrin-mediated endocytosis [14]. The endosomes containing Tf are gradually acidified, which leads to iron release; iron is reduced and transferred from the endosome to the cytosol by specialized divalent-metal transporters [15]. The ApoTf-R1 adduct is afterwards recycled back to the plasma membrane [15] where ApoTf is released into the biological fluid, ready for another iron-transport cycle. The entire process occurs in a few minutes. Therefore, Tf can be considered as a potentially important vehicle to deliver specific agents and to target practically all tissues, even across the blood-brain barrier [16–20]. The efficiency of this pathway in the targeted delivery of drugs, radionucleides, peptides, proteins and nanocarriers containing DNA vectors has been widely investigated, especially in tumor cells, where R1 is overexpressed [16–18,21].

In contrast to mammalian cells, in order to capture iron, bacteria synthesize and secrete mainly low-molecular-weight iron-specific chelating agents called siderophores [22]. These have very high affinities for iron (10^20^ ~ 10^40^ M^-1^). These bacteria can thus deprive other biological systems of iron [23–25]. However, to the best of our knowledge, the C. trachomatis genome does not encode for any known bacterial siderophore or siderophore receptor [26]. *In vitro*, in the absence of iron, *Chlamydiaceae* remain viable but not cultivable, in a persistent state, in which the RB is enlarged and non-infectious. As soon as iron is added to the culture medium, *Chlamdiaceae* growth resumes. Although the iron-acquisition system of *Chlamydiaceae* is not well understood, it was shown that in some cases Tf-R1-enriched vacuoles can fuse with the *Chlamydiaceae* inclusions [27–29], which may thus involve transferrin.

In this work, we attempt to use Tf as a Trojan horse to deliver antibiotics to C. trachomatis. We first found that, in HeLa cells, rhodamine-labeled Tf (Tf-RhB) quickly colocalizes with C. trachomatis in the bacterial inclusion. This led us to covalently graft amoxicillin onto Tf to produce a Tf-amoxicillin construct which we then tested *in vitro* and *in cellulo* as a potential vehicle capable of delivering antibiotics into the parasitophorous vacuole.

## Materials and Methods

### Ethics statement

After obtaining approval from the Institutional Review Board of the Gynecology Obstetric Service at the Antoine Béclère Hospital and the Chemistry Department at the University Paris Diderot, placentae were collected from healthy post-partum women (HIV-screened and hepatitis-C-free) from the maternity ward of the hospital. All participants were given a full explanation of the study and their written consent was obtained.

### Materials

All chemicals were of the purest available grade. They were purchased from Merck, Sigma Aldrich, Fluka, Acros and VWR. Water and glassware were prepared as described previously [12,30].

### Stock solutions

The HEPES concentration in neutral buffers was 50 mM. Final pHs were continuously measured and adjusted to between 7.2 and 8.6 with microquantities of concentrated HCl or NaOH. Tf and R1 concentrations were checked both by Bio-Rad protein assay and spectrophotometrically [12,30]. Final solutions were diluted to the required concentrations in the final buffers. All final ionic strengths were adjusted to 0.2 M with KCl [12,30]. The R1 solutions contained 10 mM CHAPS (3-[(3-cholamidopropyl)dimethylammonio]-1-propanesulfonate).

### Purification of transferrin and receptor

At least 98%-pure human-serum apotransferrin (Sigma) was further purified according to published procedures [12,30]; its purity was checked both spectrophotometrically and by urea polyacrylamide gel electrophoresis [31]. Holotransferrin (Tf), was prepared and purified as described elsewhere [30]. R1 was extracted from human placenta and purified according to published procedures [32,33]. Purity was checked by gradient sodium dodecyl sulfate polyacrylamide gel electrophoresis [33,34]. R1 was used intact without cleavage of the endo-domain. Protein concentrations were determined both spectrophotometrically and by Bio-Rad protein assay [12]. The final receptor yields varied from 3 to 5 mg per placenta.

### Fluorescent Labeling

For fluorescence microscopy assays, human transferrin was labeled by lissamine rhodamine sulfonyl chloride (RhB), as previously published [35]. Fluoresceinamine (FNH_2_) was grafted onto amoxicillin via an amide bond. The carboxylic group of the amoxicillin, which is close to the beta-lactam ring, was activated by adding microvolumes of N-hydroxysuccinamide (NHS, final concentration: 3.75 mM) and 1-ethyl-3-(3-dimethylaminopropyl)carbodiamide (EDC, final concentration: 1.5 mM). The final solution was stirred for 1 h at 5°C and then added to a large excess of FNH_2_ in HEPES buffer (50 mM) at pH 9. The mixture was then separated on permeation gel G-10 to remove the excess of both free FNH_2_ and amoxicillin.

### Grafting amoxicillin onto transferrin

To activate the carboxylic groups of Tf, microvolumes of NHS (final concentration: 3.75 mM) and EDC (final concentration: 1.5 mM) were added to 1 mL of a solution of Tf (100 μM) in 2-(N-morpholino)ethanesulfonic acid (MES, 100 mM, pH 6). The final solution was stirred for 30 min at room temperature (RT). Amoxicillin (10 mM) was dispersed in NaHCO_3_ (0.25 M) at pH 9 and then added to the protein mixture. The mixture was stirred at RT for 4 h; amide bonds were formed between the carboxylic groups in holotransferrin and the amine group of amoxicillin. Permeation gel G-50 was then used to remove the excess amoxicillin. The concentration of holotransferrin was determined both by absorption spectroscopy at 280 nm and by protein assays. Mass spectrometry was used to check the covalent bonding of amoxicillin to Tf.

### Spectrophotometric measurements

Absorption measurements were performed at 37 ± 0.5°C on a Cary 4000 spectrophotometer equipped with Peltier-thermostated cell-carriers. Fluorimetric measurements were performed at 37 ± 0.5°C on a Fluorolog 3, Horiba Jobin Yvon spectrometer equipped with a thermostated cell-carrier. Emission spectra were measured in the 300–400 nm range for an excitation wavelength (λ_ex_) of 280 nm [32]. For Tf-amox-FNH_2_, the excitation wavelength was set to 490 nm, and the emission was measured between 500 and 600 nm (λ_em_ = 523 nm). The spectra used for the static determination of the equilibrium constants were recorded at the final equilibrated state.

### Mass spectrometry

Mass spectrometry (MS) measurements were performed with an electrospray Q/TOF mass spectrometer (Q/TOF Premier, Waters) equipped with the Nanomate device (Advion). The HD_A_384 chip (5 μm I.D. nozzle chip, flow-rate range 100−500 nL/min) was calibrated before use. The Q/TOF instrument was operated in the RF quadrupole mode and data were acquired in the 400–3990 m/z range. Collision energy was set to 6 eV and argon was used as collision gas. Mass spectrometry of the intact proteins was performed after 5 min denaturation in 50% acetonitrile and 1% formic acid. Acquisition and data processing were performed with Mass Lynx 4.1 software. Deconvolution of multiply charged ions was performed by applying the MaxEnt1 algorithm. The average protein masses are annotated in the spectra, and the estimated mass accuracy is ± 2 Da. External calibration was performed with NaI clusters (2 μg/μL in 50/50 v/v isopropanol/water) in the same m/z range.

### Bacteria, cell culture and biological reagents

HeLa cells were obtained and cultured as recommended by ATCC (Manassas, VA, USA), in 75 cm^2^ tissue culture flasks for maintenance and in 24-well, 48-well or 96 well- plates for assays. C. trachomatis LGV (serovar L2) was obtained from ATCC. A stock of bacteria was prepared in HeLa cells as previously described, and stored at -80°C in sucrose-phosphate-glutamic acid (SPG) buffer (10 mM sodium phosphate [8 mM Na_2_HPO_4_ + 2 mM NaH_2_PO_4_], 220 mM sucrose, 0.50 mM L-glutamic acid) for later use [36]. Dulbecco's Modified Eagle Medium (DMEM) and fetal calf serum were purchased from Invitrogen (Carlsbad, CA, USA). Fluorescein isothiocyanate (FITC)-conjugated anti-*Chlamydia* genus antibody was from Argene (Argen Biosoft 12–114, Varhilles, France). Cell Tracker Blue CMAC (7-amino-4-chloromethylcoumarin) was obtained from Life Technologies (Saint Aubin, France).

### Localization of Tf-RhB, amox-Tf-RhB and Tf-amox-FNH_2_ in C. trachomatis-infected cells

HeLa cells were grown on coverslips for 24 h and were either infected or not with the serovar L2 of C. trachomatis at a multiplicity of infection (MOI) of 0.5. After 24 h, cells were incubated for different times and at different temperatures with Tf-RhB or amox-Tf-RhB and CMAC before being fixed in 4% neutral-buffered paraformaldehyde (PFA) for 1 h. Cells were permeabilized using methanol/ethanol (v/v) for 10 min, and coverslips were washed three times with PBS. FITC-conjugated anti-chlamydia genus antibodies were incubated for 45 min at RT in the dark. Following immunostaining, coverslips were washed three times with PBS, and subsequently counterstained for 5 min with Hoechst. Images were collected by confocal microscopy at ImagoSeine (Institut Jacques Monod, France) and further processed with Adobe Photoshop (Adobe Systems, CA, USA) or with Fiji freeware for quantitative analysis of fluorescence. For such analysis, the fluorescence of at least 30 individual cells of each group was measured.

### Treatment of C. trachomatis-infected cells by Tf-amox

HeLa cells were infected with the serovar L2 of C. trachomatis at a MOI 0.5–1 and cultured in the presence of Tf-amox at different concentrations. Cells were PFA-fixed at 24 h post-infection (P. I.) or 48 h P. I. and processed either for immunofluorescence, as described above, using an epifluorescence microscope (Leica DR) equipped with a digital camera (ORCA ER, Hamamatsu) or by transmission electron microscopy, as described below.

### Titration of infectivity

To examine the bactericidal effects of different molecules on *Chlamydia trachomatis* infectivity, we measured the infectious potential of the progeny grown in the presence of different concentrations of such molecules. Briefly, HeLa cells were cultured in 48-well plates until 70% confluence was reached. Cells were infected at a MOI of 1, for 1 hour at 37°C. After washing, the molecules (Tf, Tf-RhB, Tf-amox, Tf-amox-FNH_2_, amox) tested were added to the culture medium at different concentrations. At 48 h P.I, the cultures were harvested for measurements of progeny infectivity, as previously described [37]. This experiment was repeated three times.

### Transmission electron microscopy

HeLa cells infected with the serovar L2 of C. trachomatis (MOI 0.5–1) were fixed at the indicated time points in 2.5% glutaraldehyde in 0.1 M sodium cacodylate buffer at room temperature for 1 h. After fixation, cells were rinsed in 0.15 M sodium cacodylate buffer containing 3 mM CaCl_2_ (pH 7.4) and centrifuged at 400 g for 10 min. The pellets were re-suspended and post-fixed in 0.5% osmium tetroxide in 0.07 M sodium cacodylate buffer containing 1.5 mM CaCl_2_ (pH 7.4) at RT for 1 h, dehydrated in ethanol, and embedded in Spurr resin. Ultrathin sections were obtained using a Reicherd-Young Ultracut microtome (Leica). Sections were contrasted with 4% uranyl acetate followed by Reynold’s lead citrate and examined using a Tecnai 12 transmission electron microscope set to 80 kV and equipped with a 1k × 1k Keen View camera.

### Statistical analysis

In quantitative analyses, data are presented as the mean ± standard deviation of experiments, and p values were calculated using a Mann-Whitney Rank Sum Test.

## Results

### Transferrin colocalizes within the *Chlamydia trachomatis* inclusion

**Fig 1:**
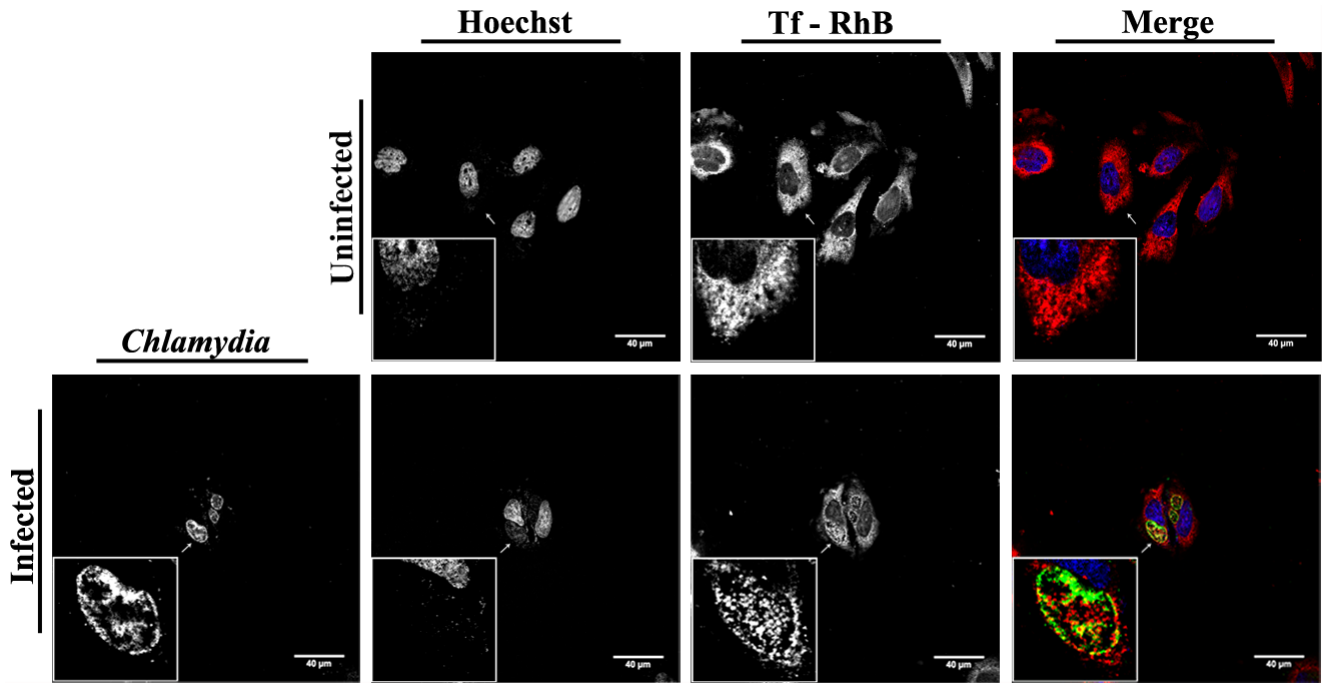
Co-localization of the transferrin and the chlamydial inclusion. C. trachomatis serovar L2-infected HeLa cells were incubated with Tf-RhB (red) for 1 h before fixation at 24 h P.I. *Chlamydia* were stained by FITC-conjugated anti-*Chlamydia* antibody (green) and host cell nuclei were stained with Hoechst (blue). Uninfected cells were seeded, processed and fixed at the same time as the infected cells. Images were collected by immunofluorescence microscopy and further processed with Adobe Photoshop.

In order to investigate the eventual involvement of transferrin in the development of bacterial inclusions, HeLa cells which were either uninfected or which had been infected with the serovar L2 of C. trachomatis for 24 h, were incubated with Rhodamine B-labeled-transferrin (Tf-RhB) for 1 h. The cells were then fixed, stained with FITC-conjugated anti-chlamydia genus antibody, counterstained with Hoechst and visualized by confocal microscopy. The results show that the transferrin is concentrated in the chlamydial inclusion in the infected cells and spreads all over the cytoplasm in uninfected cells (Fig 1). We have also determined whether the endocytosis of Tf-RhB in uninfected cells depends on the temperature. As expected, we do not observe any internalization of Tf-RhB at 4°C where endocytosis is blocked (S1A Fig). On the other hand, when HeLa cells are incubated with free Rhodamine B (RhB) at 37°C, nonspecific internalization of RhB is observed in the cytosol of the host cells (S1B Fig).

**Fig 2:**
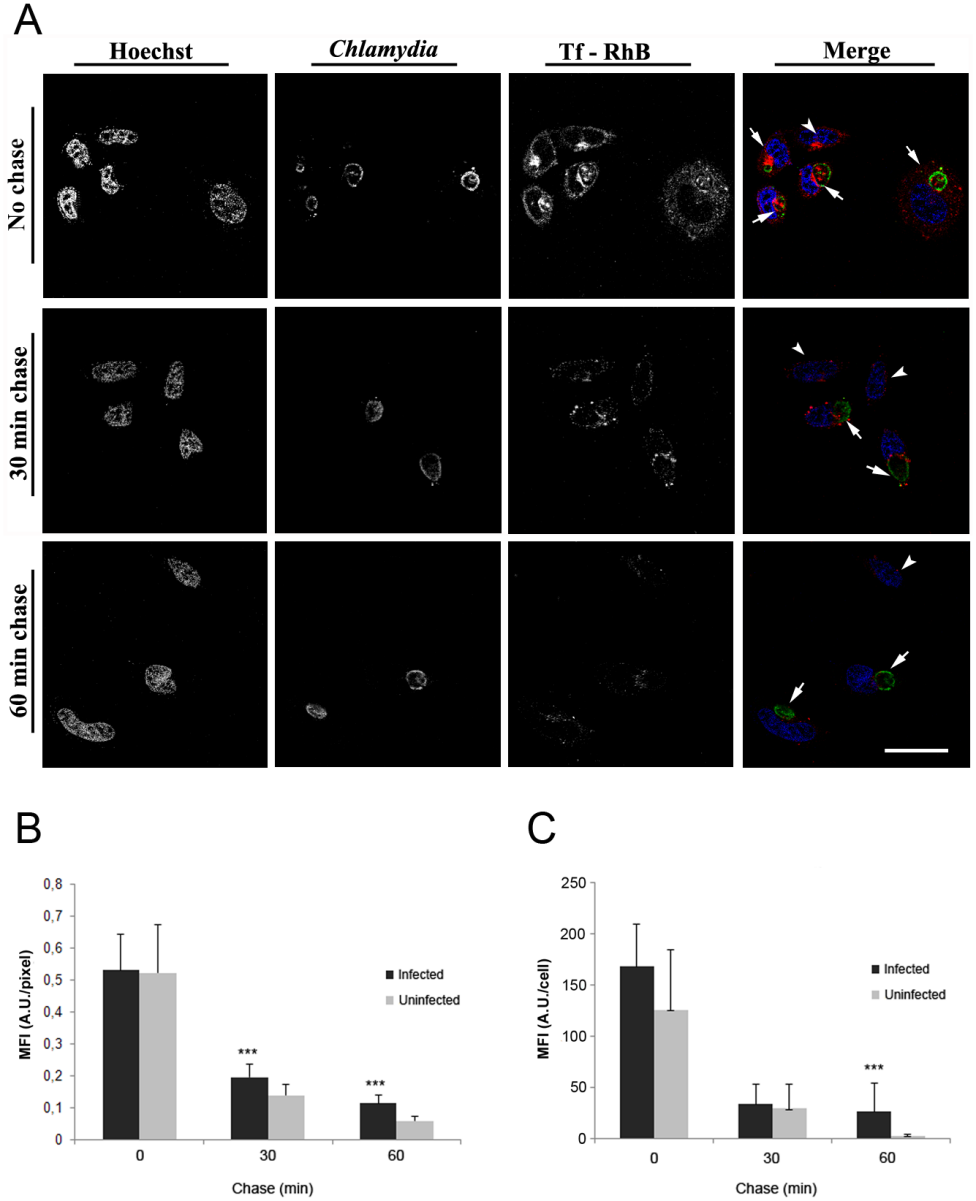
Tf-RhB recycling is delayed in *C*. *trachomatis*-infected HeLa cells. (A) HeLa cells were infected with the serovar L2 of C. trachomatis for 24 h and incubated with Tf-RhB for 1 h. Cells were allowed to recycle the Tf-RhB for 0, 30 and 60 min in the presence of unlabeled Tf before fixing and staining using anti-*Chlamydia*-FITC and Hoechst; observed under confocal microscopy (scale bar = 20 μM). (B-C) Red fluorescence (Tf-RhB) has been quantified in both infected and uninfected cells at different time of chase. Then, fluorescence intensity was related to either the number of pixels (B) or to the cell (C). *** P <0.001 vs. uninfected cells at the time of chase. Infected cells (arrows) retain more Tf-RhB than uninfected cells (arrowheads).

Cells were infected at a MOI of 0.5 for 24 h which leads to a cell infection ratio of about 35% (S2 Fig). The cells were then incubated with Tf-RhB for different time lapses (5 min– 2 h) (S2 Fig). In this case and mainly in uninfected cells, transferrin co-localized within 5 min at the perinuclear region near the microtubule organization center (MTOC). In infected cells, Tf-RhB was essentially concentrated between the nucleus and the bacterial inclusion which may be due to the compression of the nucleus and the MTOC area by the inclusion (S2 Fig). A pulse-chase experiment shows that Tf-RhB recycling is significantly delayed in C. trachomatis-infected HeLa cells (Fig 2B and 2C). Indeed, after 30 or 60 min of chase (Fig 2A), infected cells (arrows) retain more Tf-RhB than uninfected cells (arrowheads). This suggests that transferrin is rapidly transported into the cytosol of the infected cells and/or recycled.

### Conjugation of amoxicillin onto transferrin

**Fig 3:**
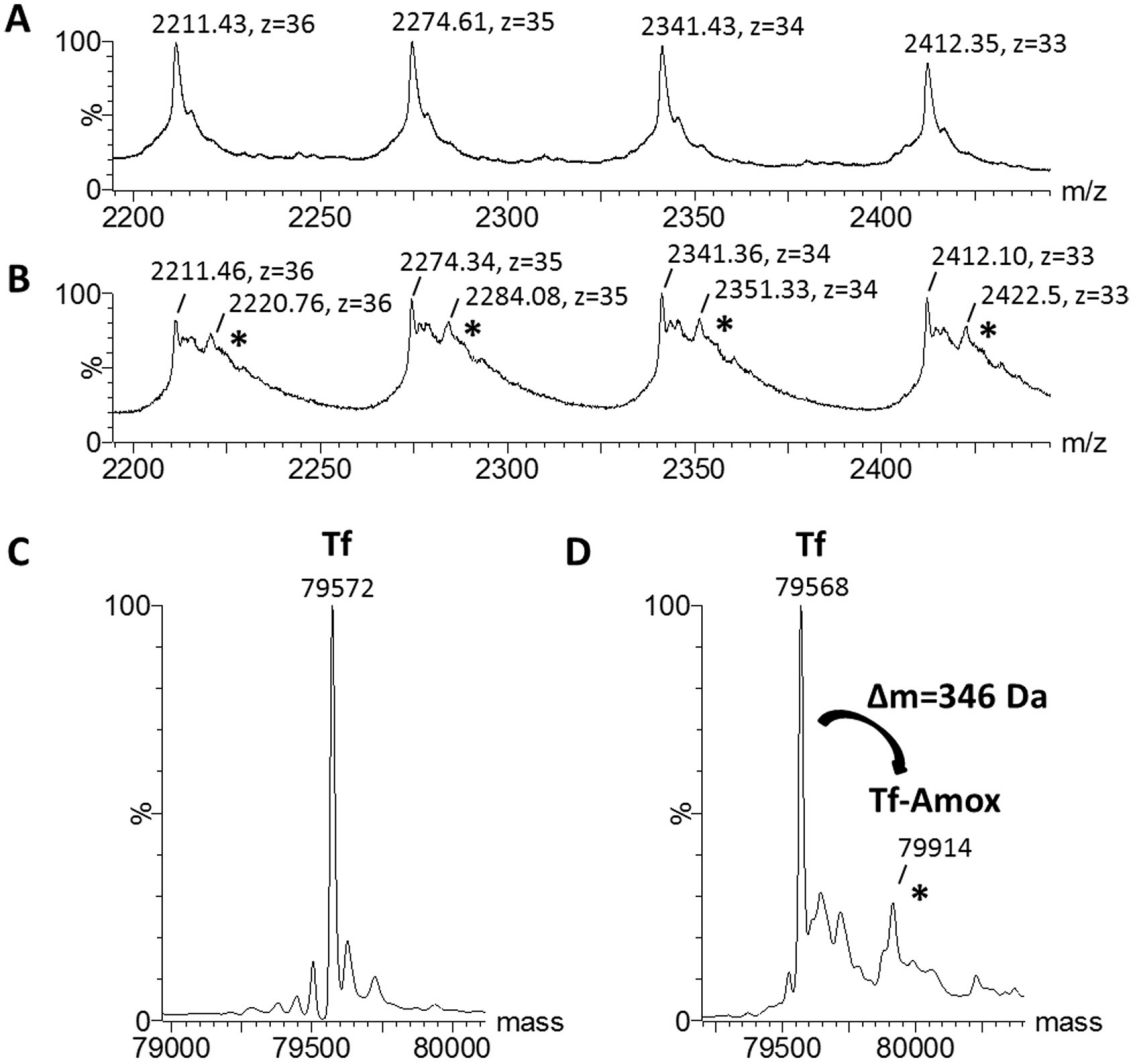
Mass spectra of Tf and Tf-amox. (A-B) Zooms of the electrospray mass spectra of transferrin before and after grafting of amoxicillin are shown in (A) and (B), respectively. Charge states z and m/z values are indicated for each peak. Mass spectra of Tf before (C) and after bonding to amoxicillin (D) obtained after deconvolution of the 400 to 3990 m/z range. Peaks with an asterisk correspond to amoxicillin covalently bound to transferrin (Tf-amox). In (D), the mass increment of (346 ± 2) Da measured for Tf-amox corresponds to the covalent binding of one amoxicillin to one transferrin. Relative abundances of Tf/Tf-amox were calculated from the ratio of the respective mass peak intensities, which were measured by integration of the peak areas under the signal curve, using the perpendicular drop method (integrated areas under peaks are depicted). Tf-amox was estimated to represent 20% of the overall protein concentration.

In order to use Tf as a carrier for targeted antibiotic delivery into the inclusion of C. trachomatis, amoxicillin was covalently grafted onto Tf. The grafting efficiency was measured by mass spectroscopy (Fig 3A and 3B). Deconvoluted electrospray mass spectra of transferrin before and after covalent cross-linking to amoxicillin are presented in Fig 3C and 3D, respectively. The presence of a mass increment of (346 ± 2) Da measured for Tf-amoxicillin (Tf-amox) is consistent with the covalent binding of one molecule of amoxicillin to one transferrin with the loss of one molecule of water and its corresponding calculated mass increment of 347 Da (Fig 3D). Relative abundance of Tf-amox to Tf was calculated by the integration of the area under the respective mass peaks. Tf-amox produced in this work represented about 20% of the overall concentration of Tf. Because the similarity of their masses (~ 79570/79917) did not permit the separation of Tf-amox from Tf, we performed all the reported experiments with a mixture of 80% Tf and 20% of Tf-amox.

### Interaction of Tf-amox with R1: Thermodynamic approach

In neutral media, holotransferrin interacts extremely well with R1. The mechanism of this interaction and the equilibrium and kinetic constants involved have been established by fluorimetric titration and chemical relaxation techniques [12]. Fluorimetric titration is a very sensitive method and is used here to analyze the interaction of Tf-amox with R1 aggregates in purely aqueous media [38]. Our experiments were, therefore, performed in the presence of CHAPS micelles where R1 is in a monomolecular form [12,32].

The addition of iron-loaded transferrin (Tf) to a solution of R1 leads to modifications in the emission spectra of the protein. This allows the measurement of the affinity constant of Tf-R1 as well as that of the Tf-RhB-R1 protein-protein adducts [12,32]. However, as shown by mass spectrometry, the concentration of Tf-amox, c_2_, is a fifth of the overall Tf concentration c_1_. Therefore, if the same conditions as those used in the literature are applied [12,32], the determination of the dissociation constant related to the interaction of Tf-amox with R1 will not be possible because c_2_ << c_1_.

**Fig 4:**
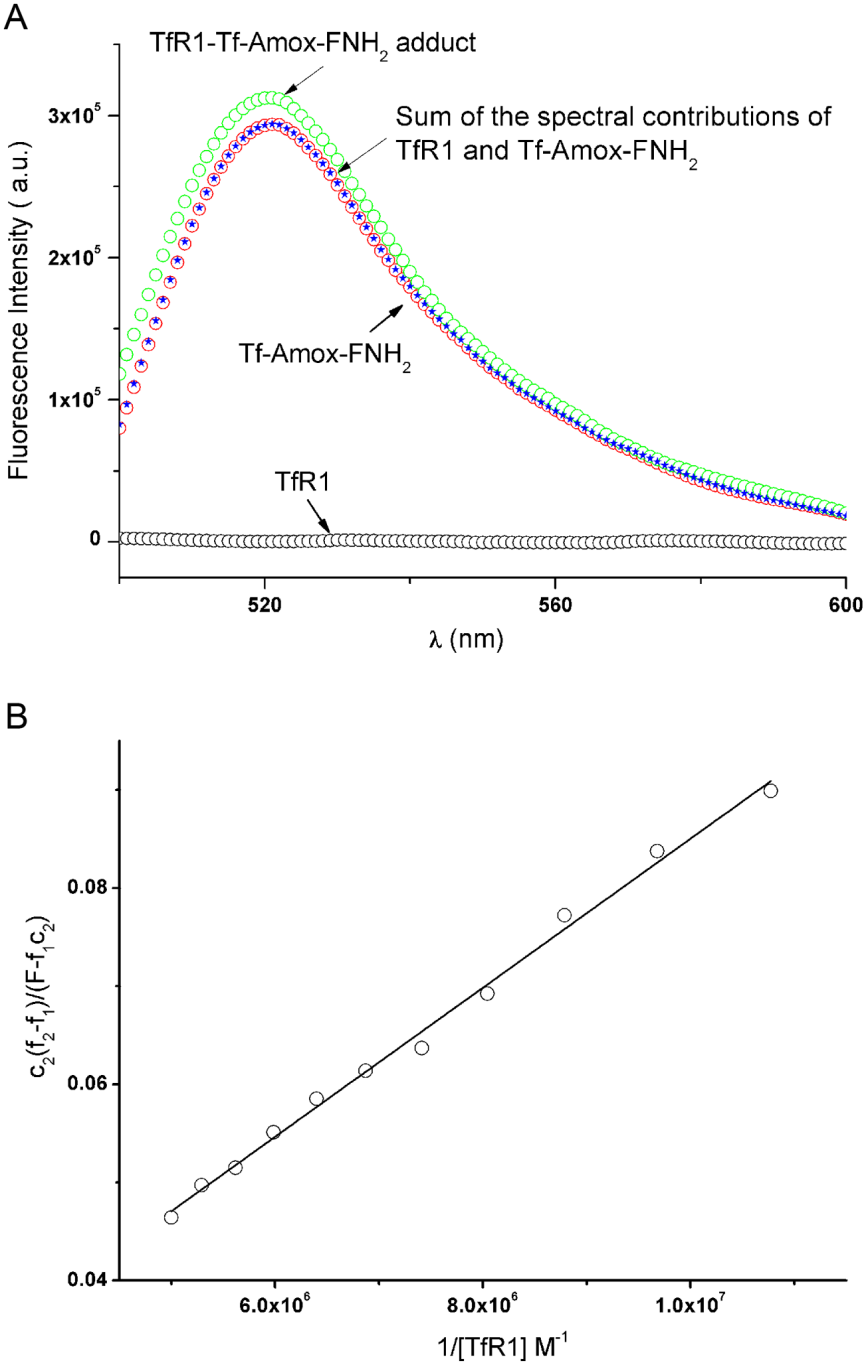
**A. Fluorescence emission spectra of Tf-amox-FNH**_**2**_**, R1 and R1 in the presence of Tf-amox-FNH**_**2**_ **after subtraction of the emission contribution of the solvent.** Values reported for an excitation wavelength λ_ex_ of 496 nm and an emission wavelength λ_em_ of 523 nm in the presence of 10 mM CHAPS with c_0_ = 2 × 10^−7^ M and c_1_ = 1 × 10^−7^ M at 37 ± 0.1°C, μ = 0.2 and pH = 7.40. **B. Plot** of $\frac{{\text{c}}_{2}\left({\text{f}}_{2}\;–{\text{ f}}_{1}\right)}{\text{F }–{\text{ f}}_{1}{\text{c}}_{2}}$ **against 1/[R]**; slope, 7.9 ± 0.2 M with c_0_ = 2 × 10^−7^ M and 0 ≤ c_1_ ≤ 1 × 10^−7^ M and ${\text{c}}_{2}=\frac{{\text{c}}_{1}}{5}$, [CHAPS] = 10 mM, and μ = 0.2 at 37 ± 0.1°C.

The excitation maximum (λ_ex_) of FNH_2_ is at 488 nm with an emission maximum at 500 nm, whereas that of Tf-amoxicillin-fluoresceinamine (Tf-amox-FNH_2_) is about 496 nm with an emission maximum of 523 nm. The excitation maxima of Tf and R1 are 280 nm, with an emission spectrum in the 300 to 400 nm region [12,32]. Therefore, when the excitation wavelength is set at 496 nm, the observed emission is only related to the Tf-amox-FNH_2_ and Tf-amox-FNH_2_-R1 species. Adding Tf-amox-FNH_2_ to R1, in the presence of 10 mM CHAPS, leads to an increase in fluorescence intensity in the 523 nm region (Fig 4A). We ascribe this variation to the molecular interaction of the receptor with Tf-amox-FNH_2_ and therefore the affinity constant between these proteins could be determined. R1 consists of two identical subunits (R), each of which interacts with Tf in its deprotonated form (Eq 1). We shall consider here the interaction of the receptor subunit (R) and Tf (Eq 2) and also that of R and Tf-amox-FNH_2_ (Eq 3) with unknown charges (Eqs 2 and 3):

$$\text{R }+{\text{ H}}^{+}\;⇄\text{ RH}$$ (1)

R + Tf   ⇄  RTf (2)

$$\text{R }+\text{ Tf}-\text{amox}-{\text{FNH}}_{2}\;⇄\text{ R}-\text{Tf}-\text{amox}-{\text{FNH}}_{2}$$ (3)

with deprotonation constant, $K_{a}=\frac{\left[\text{R}\right]\left[H^{+}\right]}{\left[\text{RH}\right]}=10\text{ nM}$, the dissociation constant of free Tf, $K_{D}=\frac{\left[\text{R}\right]\left[\text{Tf}\right]}{\left[\text{R}-\text{Tf}\right]}\;=\;2.3\text{ nM}$ [12,32] and the dissociation constant of Tf-amox, $K_{DTf-amox}=\;\frac{\left[\text{R}\right]\left[\text{Tf}-\text{amox}-\text{FNH}2\right]}{\left[\text{R}-\text{ Tf}-\text{amox}-\text{FNH}2\right]}$.

Conservation of mass gives Eqs 4–6:

$$C_{0}=\;\left[\text{R}\right]+\;\left[\text{RH}\right]+\;\left[\text{R}-\text{Tf}\right]+\;\left[\text{R}-\text{Tf}-\text{amox}-{\text{FNH}}_{2}\right]$$ (4)

$${\text{c}}_{1}\;=\;\left[\text{Tf}\right]\;+\;\left[\text{R}-\text{Tf}\right]\;+\;\left[\text{R}-\text{Tf}-\text{amox}-{\text{FNH}}_{2}]\;+\;[\text{Tf}-\text{amox}-{\text{FNH}}_{2}\right]$$ (5)

$${\text{c}}_{2}\;=\;\left[\text{R}-\text{Tf}-\text{amox}-{\text{FNH}}_{2}]\;+\;[\text{Tf}-\text{amox}-{\text{FNH}}_{2}\right]$$ (6)

The fluorescence intensity can then be expressed as:

$$\text{F }={\text{f}}_{1}\;\left[\text{Tf}-\text{amox}-\text{FTIC}\right]\;+{\text{ f}}_{2}\left[\text{R}-\text{Tf}-\text{amox}-\text{FTIC}\right]$$ (7)

where f_1_ and f_2_ are experimental parameters that relate the contributions of the Tf-amox-FNH_2_ and R-Tf-amox-FNH_2_ species to their fluorescence intensities, respectively.

Since under our experimental conditions ${\text{c}}_{2}=\frac{{\text{c}}_{1}}{5}$, Tf-amox-FNH_2_ and R-Tf-amox-FNH_2_ can be neglected, and Eqs 8, 9 and 10 can be derived from Eqs 1 to 7:

$$\left[\text{Tf}\right]\;={\text{ c}}_{1}\;–{\text{ c}}_{0}\;+\text{ α}\left[\text{R}\right]$$ (8)

$$\left[\text{R}-\text{Tf}\right]\;={\text{ c}}_{0}\;-\;\alpha \left[\text{R}\right]$$ (9)

$$\frac{{\text{c}}_{2}\left({\text{f}}_{2}\;–{\text{ f}}_{1}\right)}{\text{F }–{\text{ f}}_{1}{\text{c}}_{2}}=\;1\;+\frac{K_{DTf-amox}}{\left[\text{R}\right]}$$ (10)

Where $\text{α }=\;1\;+\frac{\left[\text{H}+\right]}{\text{Ka}}$. [R] is the positive solution of the quadratic, Eq 11:

$$\alpha \;{\left[\text{R}\right]}^{2}+\;\left({\text{c}}_{1}–{\text{ c}}_{0}\;+\;\alpha \;K_{D}\right)\left[\text{R}\right]\;–\;K_{D}{\text{c}}_{0}=\;0$$ (11)

A good linear least-squares regression of $\frac{\left({\text{f}}_{2}\;–{\text{ f}}_{1}\right)}{\text{F }–{\text{ f}}_{1}{\text{c}}_{2}}$ against $\frac{1}{\left[\text{R}\right]}$ is obtained (Fig 4B): slope, K_DTf-amox_ = 7.9 ± 0.2 nM; the uncertainty on the intercept is much too high to allow its determination. This K_DTf-amox_ value underlines a very good affinity between R and Tf-amox and is slightly higher than that obtained for free holotransferrin (2.3 nM). Therefore, *in vitro*, the Tf-amox keeps its targeting ability to deliver amoxicillin inside the bacterial inclusion.

### Tf-amox modifies the phenotype of C. trachomatis and alters the development of the bacterial inclusion

**Fig 5:**
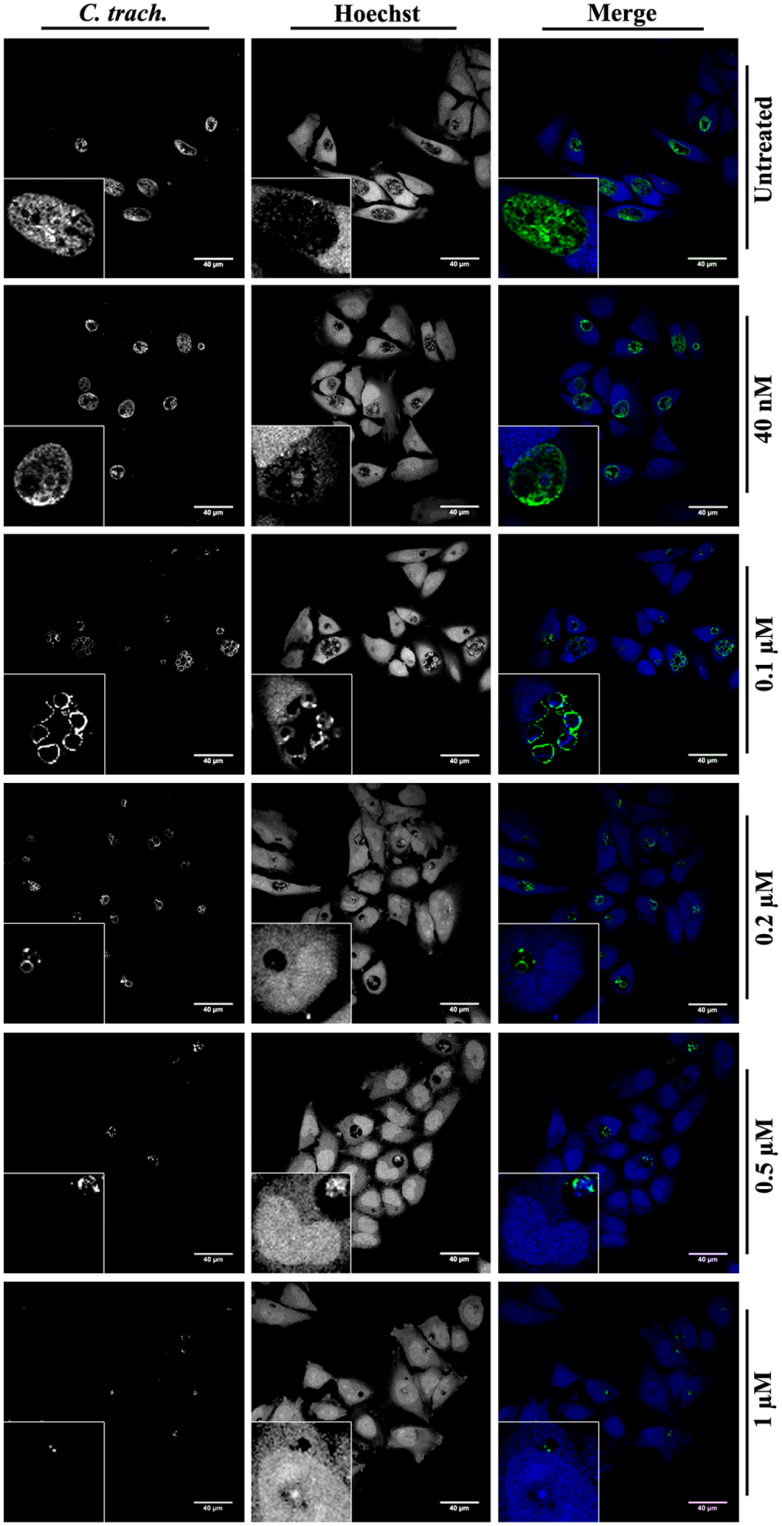
Tf-amox modifies the phenotype of C. trachomatis and alters the development of the inclusion. C. trachomatis serovar L2-infected HeLa cells were incubated with different concentrations of Tf-amox immediately after infection and were fixed at 24 h P.I. *Chlamydia* were detected using anti-*Chlamydia*-genus followed by FITC-conjugated secondary antibody (green). Cell nuclei were visualized using Hoechst (blue). Bar = 40 μM.

**Fig 6:**
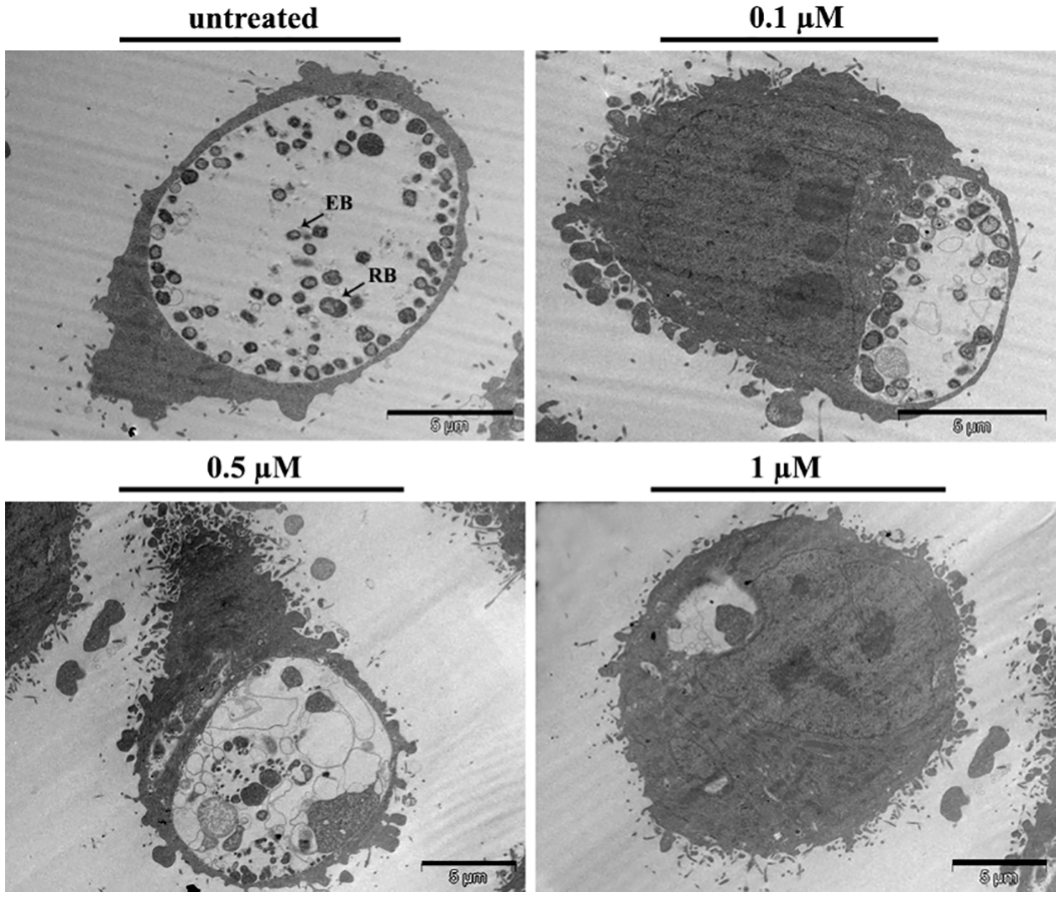
Transmission electron microscopy (TEM) of Chlamydial inclusions treated with Tf-amox. HeLa cells were infected with the serovar L2 of C. trachomatis and incubated with different concentrations of Tf-amox. At 30 h P. I., infected cells were processed for electron microscopy analysis.

The C. trachomatis development cycle is characterized by the differentiation of EBs into RBs a few hours after infection, by the exponential multiplication of RB and finally the conversion of RB into virulent EB. C. trachomatis-infected HeLa cells were incubated with different concentrations of Tf-amox, fixed at 24 h P.I. and processed for confocal microscopy analysis. We observed a Tf-amox concentration-dependent decrease in the inclusion size (Fig 5). We also observed a modification of the phenotype of the bacterial bodies starting at 0.1 μM. Such a phenotype (presence of a few swollen RB) is typical in beta-lactam (BL) treatment of *Chlamydiaceae* [39]. At higher Tf-amox concentration (0.1–1 μM), the size of the chlamydial inclusion and the number of bacteria per inclusion dramatically decrease as compared to controls (Fig 5). In another experiment, amoxicillin was grafted onto RhB-labeled transferrin (Tf-RhB) and infected cells were treated using the protocol described above. The results showed a localization of amox-Tf-RhB on the periphery as well as in the inclusion. This can be due to transferrin recycling which is required for iron uptake by the cell and therefore for its survival. However, the appearance of the BL phenotype of the bacteria implies that the Tf-grafted antibiotic is targeted towards the inclusion (S3 Fig). Moreover, electron microscopy analysis performed at 30 h P.I. confirmed the abnormal swelling of RB and the appearance of membranous vesicles free of biological material in the inclusions of the infected cells treated with at least 0.1 μM of Tf-amox (Fig 6). At 1 μM of Tf-amox, the inclusions become very small and contain one or two dilated bacteria.

### Tf-amox has a bactericidal effect on C. trachomatis at lower concentration than free amoxicillin

**Fig 7:**
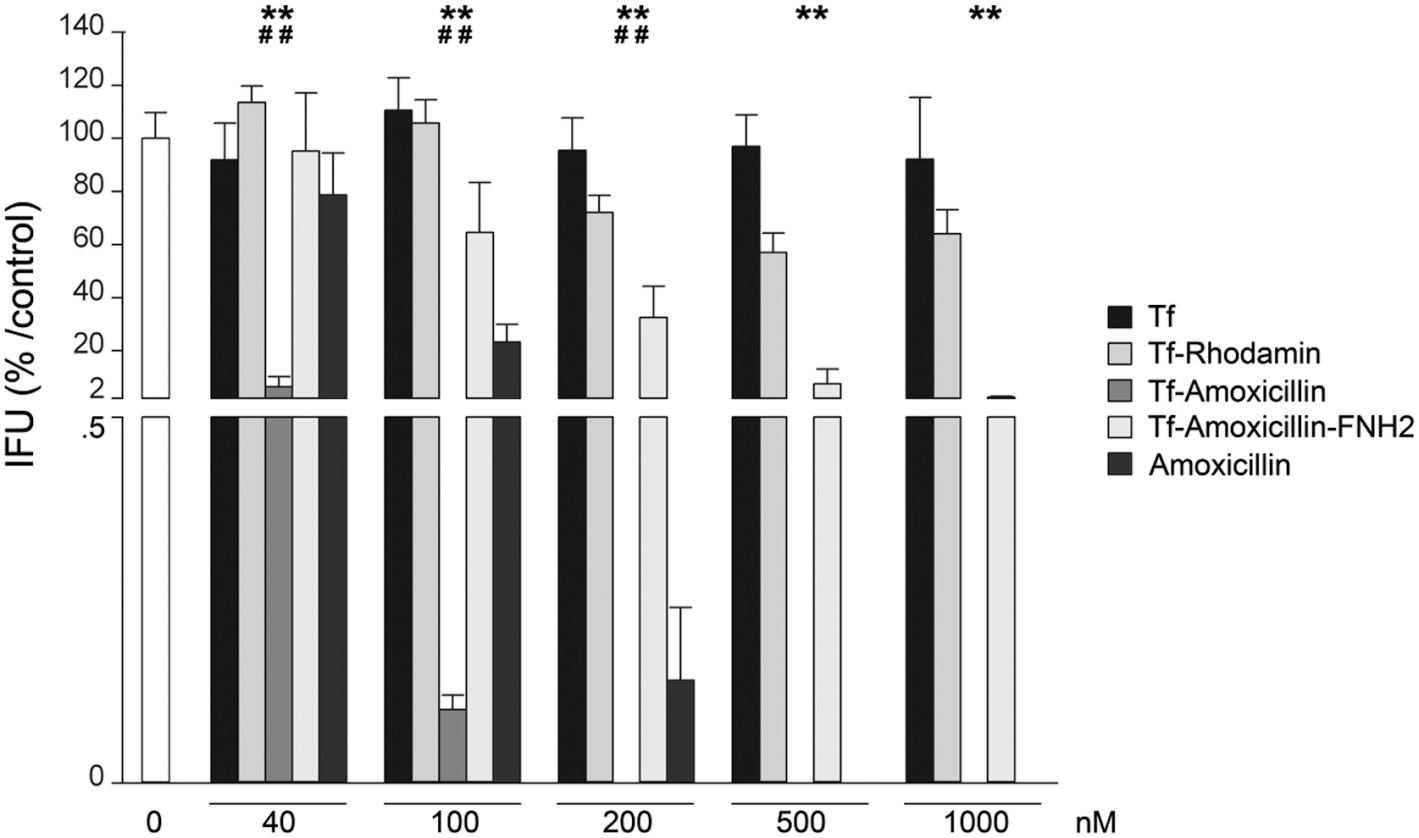
Inhibitory effects of Holotransferrin (Tf), Tf-RhB, Tf-amox, Tf-amox-FNH_2_ and amoxcillin on C. trachomatis infectious capacity *in cellulo*. Statistically significant differences are noted as follows: **p = 0.002 compared to Tf-amox-FNH_2_, ^##^*p* = 0.002 compared to amoxicillin.

Since Tf-amox shows an effect on the phenotype of C. trachomatis and on the size of the inclusion, we measured the bactericidal effect of this molecule on C. trachomatis as compared to free amoxicillin, Tf-amox-FNH_2_ (beta lactam ring blocked), Tf and Tf-RhB (Fig 7). We showed that, at the lower concentrations tested (40–200 nM), the anti-chlamydial effect of Tf-amox was significantly stronger than that of free amoxicillin. We also showed that the bactericidal activity of Tf-amox is reached at between 100 and 200 nM, whereas this effect is reached at between 200 and 500 nM for free amoxicillin.

Overall, we can conclude that part of the cellular Tf is addressed to the Chlamydial inclusion and that Tf-amox is targeted towards the inclusion where it has a bactericidal effect at lower dosage than free amoxicillin. All these results suggest that transferrin can be used as an effective carrier for targeted antibiotic delivery to *Chlamydiaceae* and maybe other intracellular bacteria.

## Discussion

*Chlamydia trachomatis* development is highly dependent on iron. It is well established that this bacterium enters a persistent state when infected cells are incubated with an iron chelator, such as desferroxamine [39]. The withdrawal of the chelator leads to resumption of the bacterial cycle. However, iron-acquisition pathways of C. trachomatis are not well understood. Indeed, the genome of C. trachomatis does not contain genes encoding for the enzymes required for siderophore synthesis and/or those required for receptors of eukaryotic iron-binding proteins [40]. It would appear that the iron requirements of *Chlamydiaceae* are fulfilled through highly effective strategies in which the bacterium highjacks iron from the biosynthetic pathways, the organelles and the traffic vesicles of the host cell [41]. Among the latter, it has often been shown that vesicles bearing R1 are located in the vicinity of or inside the inclusion of the *Chlamydia* species. This is the case of the monocytic cell Thp1 infected with *C*. *psitacci* [42]. The receptor was also shown to be at least partly localized in the inclusions of L2-infected HeLa cells [27]. In 1999 and 2001, Al Younes *et al*. showed a localization of R1 in a *Chlamydia pneumoniae* inclusion at both 20 h P.I. and 40 h P.I. but not at 70 h P. I. This specific localization is stronger in iron-starved infected cells than in uninfected controls [28,43]. Furthermore, although the expression of R1 does not appear to be modulated by *Chlamydia* infection, it seems that the fusion of bacterial inclusions with the vesicles carrying R1 is required for bacterial development [29,43,44]. However, there is no direct evidence for Tf delivery into the chlamydial inclusion.

Our *in cellulo* study shows that Tf-RhB is endocytosed within minutes into HeLa cells infected by the serovar L2 of C. trachomatis; it accumulates in 30 minutes within the bacterial inclusion and seems to be co-localized with the bacteria.

These results might seem at odds with other studies in which Tf was excluded from the chlamydial inclusion. In 1996 and 2003, Scidmore *et al*., incubated *Chlamydia* L2-infected HeLa cells for 15 minutes with Tf-HRP in a serum-free medium before fixation. The labeling observed by transmission electron microscopy was predominantly outside the inclusion but, nevertheless, a few labeled vesicles were also localized inside [44,45]. Another study, dealing with HeLa cells infected with the serovar E of C. trachomatis for 24 h and afterwards incubated with Tf-HRP for 1 h, showed weak staining inside the inclusion. However, in this study Tf-HRP was used at a concentration of 10^−8^ M (about 800 μg.L^-1^) [46]. Using fluorescent transferrin, Rzomp *et al*. showed a fusion of Rab1-, Rab4- and Rab11-positive vesicles with the inclusion of the serovar L2 of *Chlamydia trachomatis* at 18 h. P.I. but failed to detect Tf in the inclusion [47].

The discrepancies between these results and ours may be due to the fact that our experiments were performed with a higher concentration of Tf-RhB. Nevertheless, this concentration (250 μg.mL^-1^) is still 10 times lower than that of serum transferrin in the bloodstream (about 2.5 g.L^-1^) [48]. The differences may also be the result of the incubation time with the fluorescent probe before fixation. In our work, it was rare to observe Tf staining inside the bacterial inclusion before 30 min P. I. with Tf-RhB. Finally, the differences may also arise from the *Chlamydiaceae* species used (*Chlamydia pneumoniae* or a serovar of C. trachomatis) and/or from the post-infection time at which the infected cells were incubated with Tf-RhB. On the other hand, *Chlamydiaceae* may acquire iron in a narrow P. I. time window. In our study, we confirmed the pulse-chase experiments of Van Ooij *et al*., showing that Tf recycling is delayed in HeLa cells infected by the serovar L2 of C. trachomatis [27]. This recycling rate has been correlated with the development and growth of the bacterial inclusion [29].

Transferrin receptor-mediated endocytosis has been widely used for the delivery of anticancer drugs and therapeutic genes, primarily for targeting proliferating malignant cells overexpressing R1 [49]. Antibacterial effects of antibiotics in non-covalent interaction with ovotransferrin have also been investigated with a certain degree of success [50]. In our case, the antibiotic chosen was amoxicillin. Amoxicillin is not very commonly used for the treatment of *Chlamydia* (except in pregnant women), but, as a beta-lactam, it induces a particular phenotype of the bacteria, easily identifiable in microscopy [39]. In Tf-amox, the antibiotic is covalently bound to the protein with, a ratio of Tf-amox/Tf ≈ 0.2. On the other hand, we showed that the affinity of Tf-amox for R1 is practically identical to that of Tf, which implies the recognition of Tf-amox by the receptor and its internalization by receptor-mediated endocytosis. Again, as Tf-amox represents only 20% of the overall transferrin concentration, its scarcity should limit its internalization as compared to Tf. Nevertheless, Tf-amox was found to have significantly higher bactericidal activity on C. trachomatis than free amoxicillin.

The co-localization of Tf with C. trachomatis in the bacterial inclusion led us to envisage that R1-mediated iron acquisition constitutes a possible pathway for the delivery of drugs, such as antibiotics. We, therefore, assume that Tf facilitates the delivery of amoxicillin from the bulk to the bacteria. Indeed, the targets of the BL are the bacterial enzymes responsible for the synthesis of a peptidoglycan-like-molecule involved in bacterial division [51,52], Our results show that Tf probably routes grafted amoxicillin into the bacteria and that this amoxicillin keeps its antibiotic activity by inhibiting the enzymes involved in the peptidoglycan-like-molecule synthesis.

## Conclusion

Our work suggests that Tf could be involved in the acquisition of iron by C. trachomatis. Thus, the Tf-R1 iron-acquisition pathway can be envisaged to specifically target *Chlamydia* inside the infected cell. Grafting an antibiotic, such as amoxicillin, onto transferrin does not interfere with the recognition of the protein by its receptor. Despite the rather low grafting yield (20%), Tf-amox undergoes efficient internalization by receptor-mediated endocytosis, which allows the antibiotic to reach the bacteria and to have higher anti-*Chlamydia* effect than free amoxicillin.

These results show for the first time that the iron-acquisition pathway of mammals can be efficiently used in the treatment of *Chlamydia*. To further complete these findings, antibiotic grafting should be improved and other antibiotics should be tested. We also hope that this will provide a new approach to avoid multidrug resistance in cancers and intracellular bacterial infections [9].

## Supporting Information

S1 Fig Localization of the Tf-RhB (A) and RhB (B) in HeLa cells incubated at 37°C and 4°C. HeLa cells were seeded for 24 h and incubated for different times and at different temperatures with either Tf-RhB (A) or RhB (B) and with Cell Tracker Blue CMAC (7-amino-4-chloromethylcoumarin). Cells were fixed after 15 min, 30 min or 60 min before being stained with Hoechst (blue). Images were collected by confocal microscopy and further processed with Adobe Photoshop.
(TIF)

S2 Fig Time-dependent localization of transferrin in Chlamydia-infected- and uninfected cells. At 24h P.I., *C*. *trachomatis* serovar L2-infected HeLa cells were incubated with Tf-RhB (red) for different times and with Cell Tracker Blue CMAC. Cells were fixed and stained using a FITC-conjugated anti-*Chlamydia* genus antibody (green) and with Hoechst (blue). Uninfected cells were seeded and fixed at the same time as the infected cells.
(TIF)

S3 Fig Localization of amox-Tf-RhB into the chlamydial inclusion. C. trachomatis serovar L2-infected HeLa cells were incubated with amox-Tf-RhB and CMAC and fixed at 24 h P. I. *Chlamydia* were stained using FITC-conjugated anti-*Chlamydia* genus antibody (green); the host cell nuclei were stained with Hoechst (blue). Bar = 40 μM. Arrows show amox-Tf-RhB in swollen bacteria. The bottom figure shows an enlargement of the top panel. The periphery of the chlamydial inclusions is drawn in white. Bar = 10 μM.
(TIF)
