# Supplementary figures and images for: Targeted Delivery of Amoxicillin to C. trachomatis by the Transferrin Iron Acquisition Pathway
Source: PLoS One. 2016 Feb 26;11(2):e0150031. doi: 10.1371/journal.pone.0150031 (PMC4768884; doi:10.1371/journal.pone.0150031)

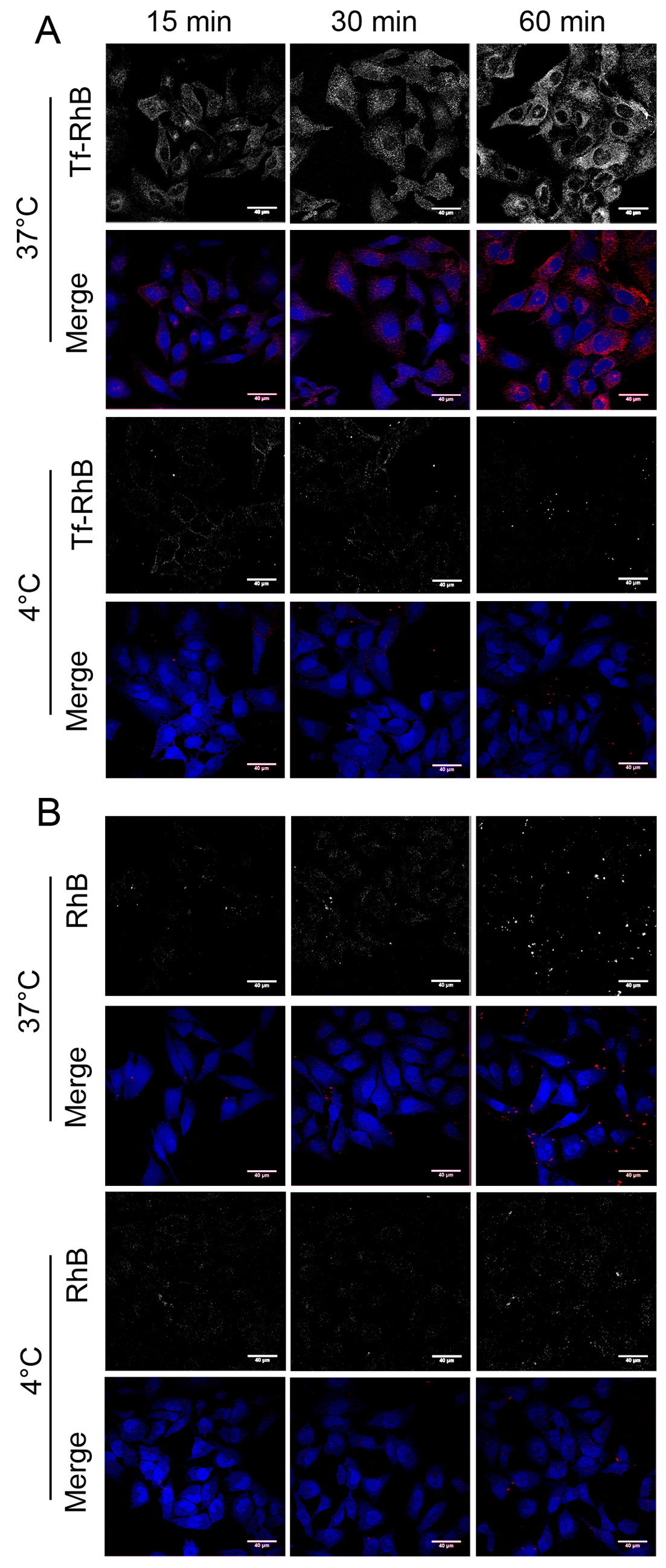

Supplement: S1 Fig — HeLa cells were seeded for 24 h and incubated for different times and at different temperatures with either Tf-RhB (A) or RhB (B) and with Cell Tracker Blue CMAC (7-amino-4-chloromethylcoumarin). Cells were fixed after 15 min, 30 min or 60 min before being stained with Hoechst (blue). Images were collected by confocal microscopy and further processed with Adobe Photoshop. (TIF) [file pone.0150031.s001.tif]

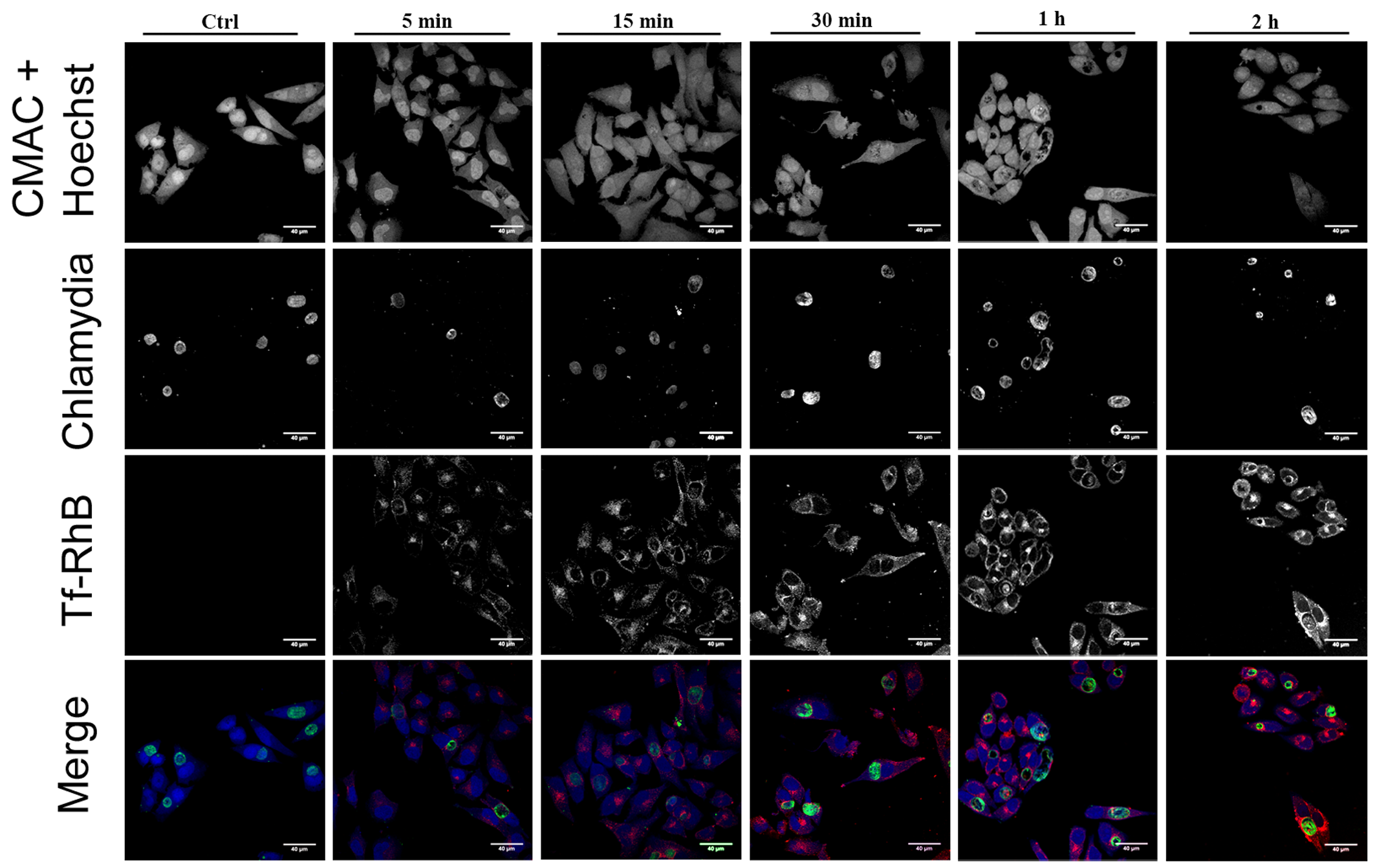

Supplement: S2 Fig — At 24h P.I., C. trachomatis serovar L2-infected HeLa cells were incubated with Tf-RhB (red) for different times and with Cell Tracker Blue CMAC. Cells were fixed and stained using a FITC-conjugated anti-Chlamydia genus antibody (green) and with Hoechst (blue). Uninfected cells were seeded and fixed at the same time as the infected cells. (TIF) [file pone.0150031.s002.tif]

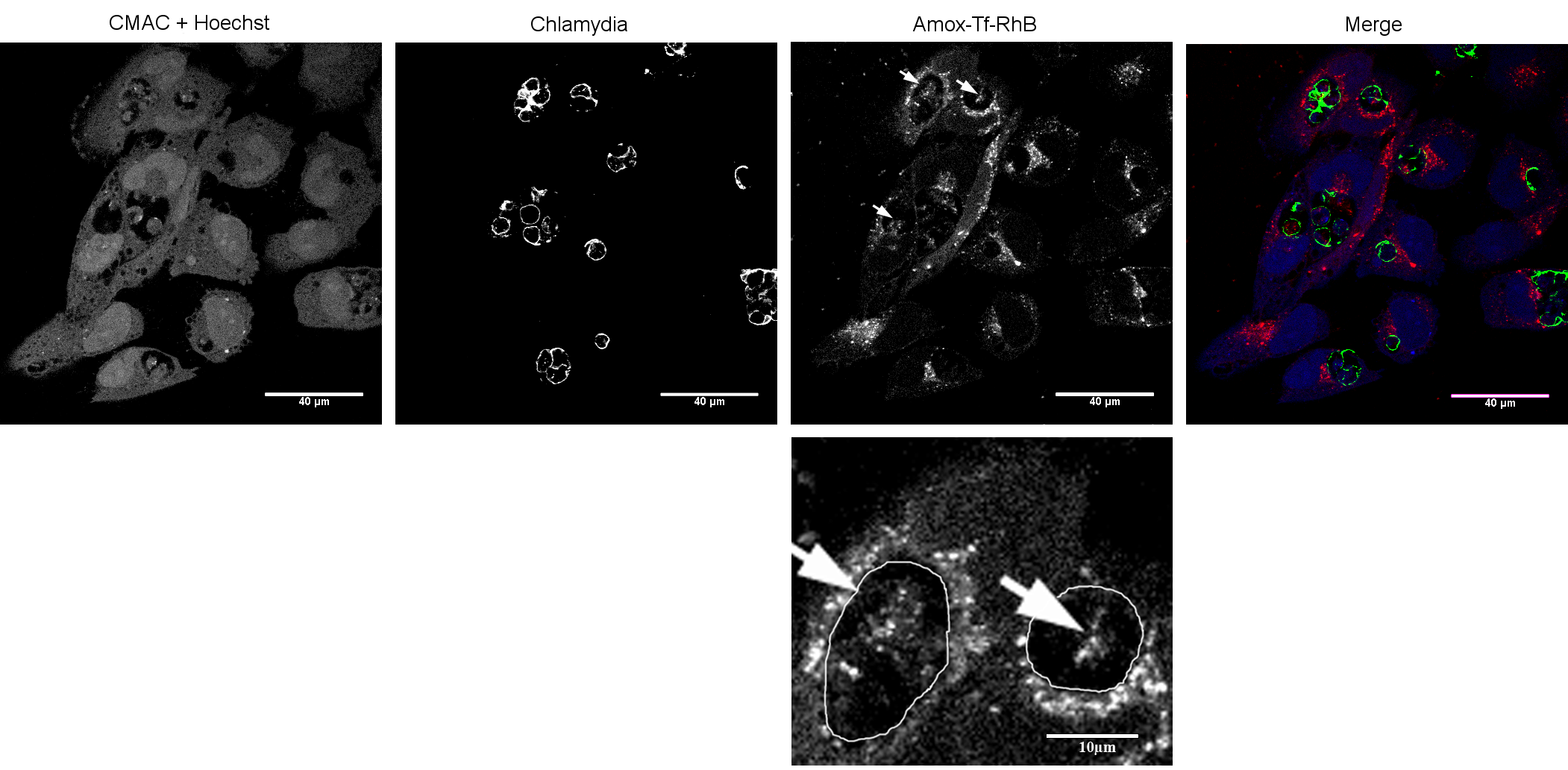

Supplement: S3 Fig — C. trachomatis serovar L2-infected HeLa cells were incubated with amox-Tf-RhB and CMAC and fixed at 24 h P. I. Chlamydia were stained using FITC-conjugated anti-Chlamydia genus antibody (green); the host cell nuclei were stained with Hoechst (blue). Bar = 40 μM. Arrows show amox-Tf-RhB in swollen bacteria. The bottom figure shows an enlargement of the top panel. The periphery of the chlamydial inclusions is drawn in white. Bar = 10 μM. (TIF) [file pone.0150031.s003.tif]
